# Buffy Coat Processing Impacts on Monocytes’ Capacity to Present Lipid Antigens

**DOI:** 10.3390/biomedicines11030833

**Published:** 2023-03-09

**Authors:** Inês Mondragão-Rodrigues, M. Fátima Macedo

**Affiliations:** 1 Cell Activation and Gene Expression Group, Instituto de Investigação e Inovação em Saúde (i3S), University of Porto, 4200-135 Porto, Portugal; 2 Department of Medical Sciences, University of Aveiro, 3810-193 Aveiro, Portugal

**Keywords:** Buffy Coat, iNKT cells, CD1d, monocyte, antigen presentation

## Abstract

Buffy Coats, generated from a blood donor’s whole blood bag unit, are commonly used in biomedical research as a source of leukocytes due to the high number of cells that can be recovered from each Buffy Coat. Buffy Coats are leukocyte-enriched residual units obtained by centrifugation of whole blood. At the blood bank, blood can be processed using two different protocols according to the time interval between blood collection and processing. When blood collection and processing occur on the same day, it gives rise to Fresh Blood Buffy Coats. Alternatively, if blood processing only happens on the day after blood collection, Overnight Blood Buffy Coats are created. In this study, we aimed to address whether these two different Buffy Coat-processing protocols could differently impact monocyte function as antigen-presenting cells. For this purpose, we analyzed in the same experiment monocytes isolated from Fresh Blood and from Overnight Blood Buffy Coats. We assessed lipid antigen presentation by CD1d to invariant Natural Killer T (iNKT) cells. CD1d is a non-polymorphic MHC class I-like protein, which facilitates the study of antigen presentation among allogeneic samples. The results show that monocytes from Fresh Blood Buffy Coats have a better capacity to present antigens by CD1d, and consequently to activate iNKT cells, when compared to monocytes from Overnight Blood Buffy Coats. The differences observed were not explained by disparities in monocyte viability, CD1d expression, or basal activation state (monocyte expression of CD40 and CD80). Buffy Coats are a valid source of blood cells available daily. Hence, the type of protocol for Buffy Coat processing should be carefully considered in day-to-day research, since it may lead to different outcomes.

## 1. Introduction

**Figure 1:**
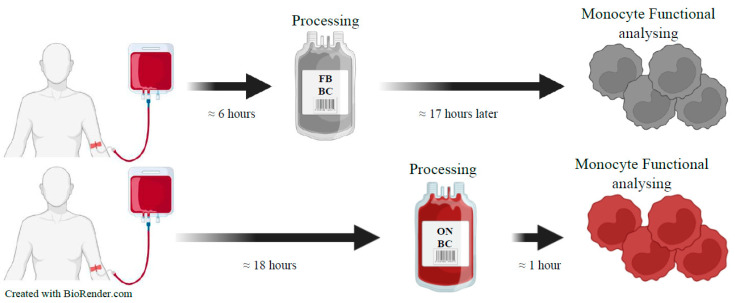
Scheme of the two different timing protocols of processing whole 450 mL blood to obtain BC and respective waiting times. For Fresh Blood BC, the collection and processing of the whole blood occurs on the same day. Fresh Blood BC must wait for the following day to be used in experiments. As for the Overnight Blood BC, the whole blood is only processed one day after the blood collection. The Overnight Blood BC is used for assays on the same day as it is processed. FB BC: Fresh Blood Buffy Coat; ON BC: Overnight Buffy Coat.

At the blood bank, the processing of whole blood, a 450 mL donation, originates red cells, platelet units, plasma, and a waste product also known as Buffy Coat (BC). BCs may be obtained by two different protocols according to the time interval between blood collection and processing: (1) Fresh Blood BC, in which the processing of the whole blood is performed on the same day as the blood donation and (2) Overnight Blood BC, in which the whole blood is processed in the day after the blood collection (Figure 1).

The BC is a reliable, consistent, and widely used source of leukocytes, including monocytes, for biomedical research purposes. In healthy donors, reference ranges of different cell populations percentages present in BCs are: granulocytes 42–77%; lymphocytes 20–44%, and monocytes 2–9.5% [1].

Monocytes are instrumental for several events that happen in the immune system. They are the primary type of mononuclear phagocyte in blood, and they are traditionally looked at as a source of tissue macrophages. However, recent studies underline a more complex role for monocytes in innate and adaptative immunity, particularly as antigen presenting cells [2].

Monocytes act as antigen-presenting cells to both conventional and unconventional T cells, including invariant NKT (iNKT) cells. iNKT cells are unconventional T cells that are reactive to the nonclassical major histocompatibility complex (MHC) class I-like antigen-presenting molecule CD1d and are characterized by the expression of a semi-invariant TCR, which, in humans, combines a Vα24Jα18 chain paired with a Vβ11 chain [3,4]. CD1d is a non-polymorphic molecule facilitating the allogenic use of iNKT cells in immunotherapies [4,5]. iNKT cells are an important lymphocyte population that sense a range of endogenous and exogenous lipid antigens presented by CD1d. These cells recognize glycolipid antigens, namely α-Galactosylceramide (α-GalCer), which is the prototype lipid antigen for iNKT cells. Other endogenous and exogenous iNKT cell lipid antigens have been discovered [3,4]. The lipid α-Gal-(α1-2)-α-GalCer (α-GalGalCer) becomes an iNKT cell antigen upon internalization and galactose removal by α-galactosidase A in the lysosome [6]. Upon stimulation, iNKT cells respond rapidly to both innate signals and TCR engagement by promptly producing large amounts of cytokines, which are important in the modulation of several immune responses against infection and cancer [4,5].

Monocytes are frequently used in immunological and pharmacological studies, diagnostics, and clinical trials. These assays depend on the physiological state of monocytes. Pre-analytical factors, such as blood collection technique (different tubes and anticoagulants), blood storage, monocyte isolation technique, and freezing may directly interfere with monocyte properties. For instance, monocyte CD14 expression is affected by the type of anticoagulant used in blood collection [7]. Few studies have been conducted to assess the effect of pre-analytical factors on monocyte properties. Longer delays in blood processing lead to increased granulocyte contamination of Peripheral Blood Mononuclear Cells (PBMCs), a decrease in NK cells’ ability to degranulate, and low cell recovery and affects PBMCs’ proteomic patterns [8,9].

In the present study, we investigated whether the antigen-presentation capacity of monocytes to present lipid antigens to iNKT cells is influenced by different BC processing protocols.

## 2. Materials and Methods

Harvest of the whole blood for both Fresh Blood BC and Overnight Blood BC was completed on the same day from different donors. Whole blood collection units (450 mL) were maintained at room temperature for approximately 6 h for the units that would give rise to Fresh Blood BC and 18 h for the units that would generate Overnight Blood BC. Afterwards, the blood units, from different subjects, were processed into the different products (Red blood cells, plasma, platelets, and BC) at the hospital using the Reveos^®^ system by centrifugation at 2600 or 2900 rpm (depending on the blood product desired) for 7 min. Then, PBMCs were isolated at the same time, in our laboratory, from the BC obtained by the two different protocols: Fresh Blood BC and Overnight Blood BC. 

All the experimental protocols were performed using BC kindly provided by the Blood Bank of the Centro Hospitalar Universitário de São João, Porto (Portugal). 

### 2.1. Peripheral Blood Mononuclear Cells (PBMCs) Isolation

PBMCs were isolated from the BC using Histopaque-1077^®^ (Sigma-Aldrich, St. Louis, MO, USA) density centrifugation following the manufacturer’s instructions. After centrifugation, the PBMCs ring, located between the plasma and Histopaque-1077^®^, was recovered, followed by washing with PBS. Incubation with Red Blood Cell Lysis Buffer (BioLegend, San Diego, CA, USA) was performed to lysate remaining erythrocytes, and, after wash and cell count, PBMCs were used for CD14^+^ cells isolation.

### 2.2. Monocytes Isolation through Positive Selection

Monocytes were isolated by positive selection with anti-CD14 magnetic beads using the MACS cell separation system (MiltenyiBiotec, Cologne, Germany) following the manufacturer’s instructions. Succinctly, the PBMCs were incubated with magnetic beads coated with the anti-CD14 antibody and passed through a magnetic column. The magnetic force existing between the beads and the support keeps the labeled cells from eluting. The cells that bind to the beads (monocytes) remain in the column, while non-labeled cells are eluted. When the column is removed from the magnetic support, the CD14^+^ cells can be eluted. CD14^+^ cells were used, after purification, for flow cytometry analysis and lipid antigen presentation assays.

### 2.3. iNKT Cell Line Culture Maintenance and Re-Stimulation

An iNKT cell line was generated and maintained through re-stimulations as previously described [10]. In short, the iNKT cell line was generated by culturing PBMCs with 100 ng/mL of α-GalCer (KRN7000) and 100 U/mL of IL-2. These PBMCs, and consequently the iNKT cell line, were derived from a healthy individual. After 11 days, CD1d-PBS57 tetramer^+^ CD3^+^ cells were sorted using FACSAria (BD Biosciences). iNKT cells were maintained in culture at 37 °C, 5% CO_2_ by periodic re-stimulation with irradiated PBMCs in the presence of 1 μg/mL of PHA (Thermo Fisher Scientific, Waltham, MA, USA), and 100 U/mL of IL-2.

### 2.4. Flow Cytometry

Monocyte purity and the basal state of activation of cells were assessed by flow cytometry using the following anti-human monoclonal antibodies: CD14 (61D3, eBiosciences, San Diego, CA, USA), CD1d (51.1, Biolegend San Diego, CA, USA), CD80 (2D10, Biolegend, San Diego, CA, USA), and CD40 (5C3, BioLegend San Diego, CA, USA). Monocyte viability was assessed by flow cytometry using Fixable Viability Dye (FVD—eBiosciences, San Diego, CA, USA). Cells were stained with the previously mentioned antibody cocktails diluted in PBS/2%FBS/1mM EDTA/0.01%NaN_3_ (flow cytometry solution) for 20 min, at 4 °C, in the dark. After staining, cells were washed with flow cytometry solution and then fixed with PBS 1% formaldehyde. The purity of the iNKT cell line was tested using CD1d-PBS57 tetramers (NIH Tetramer Core Facility, Emory University, Atlanta, GA, USA) and anti-human CD3 (UCHT1, eBioscience, San Diego, CA, USA). Cells were acquired in a FACS Canto II (BD Biosciences, San Diego, CA, USA) using the BD FACS Diva^TM^ software (BD Biosciences). Data analysis was performed with FlowJo^R^ v10 (FlowJo LLC, Ashland, OR, USA).

### 2.5. Lipid Antigen Presentation Assays

Monocytes were cultured with α-Galactosylceramide (α-GalCer) (at 3.1, 12.5, or 50 ng/mL, KRN700, Sigma) or Gal(α1-2) αGalactosylceramide (α-GalGalCer or PBS 18, provided by Paul B. Savage from Brigham Young University, Provo, UT, USA; PBS 18 stands for the internal classification of the lipid synthetized by Paul B. Savage) (at 50, 150, or 300 ng/mL) and Phytohemagglutinin (PHA, Thermo Fisher Scientific at 1 μg/mL). α-GalGalCer was first dissolved in PBS 0.5% Tween 20 and then diluted in non-supplemented RPMI to have a maximum of 1% vehicle in culture. α-GalCer was resuspended in PBS and directly diluted in non-supplemented RPMI. After 4 h, an iNKT cell line was added, and cells were cocultured for 40 h at 37 °C, 5% CO_2_. After 40 h, supernatants were collected for cytokine production determination by ELISA. The following antibody pairs from Biolegend were used: purified anti-human GM-CSF (BVD2-23B6) and purified anti-human IL-4 (8D4-8).

### 2.6. Statistics

An unpaired *t*-test (normal distribution) was used to compare two groups. *p*-values lower than 0.05 were considered statistically significant. Along with the comparison of the raw data, and due to the inter-experimental variation, comparison was also performed after normalization of the values of cytokine production for each independent experiment. The values of cytokine production were normalized, considering 100 as the highest cytokine production value within each experiment for the chosen antigen concentration. All the analyses were performed using GraphPad Prism software v6 (GraphPad Software Inc., San Diego, CA, USA).

## 3. Results and Discussion

### 3.1. Monocytes Isolated from Fresh Blood Buffy Coats Have a Better Capacity to Activate iNKT Cells than Monocytes Isolated from Overnight Blood Buffy Coats

Herein we investigate whether the different BC-processing protocols influence monocyte antigen presentation capacity. In the same experiment, the capacity of monocytes isolated from Fresh Blood BC and Overnight Blood BC to activate iNKT cells was compared. As described in Figure 1, for Fresh Blood BC, whole blood was processed within 6 h after blood donation, and BCs were left to rest for approximately 17 h before monocyte isolation and analysis. For Overnight Blood BC, whole blood was processed approximately 18 h after blood donation, and monocyte isolation performed within 1 h after BC production.

Anti-CD14 beads were used to isolate monocytes by positive selection. The monocytes were incubated using a human iNKT cell line and α-GalCer or α-GalGalCer lipid antigens. Monocytes from Fresh Blood BC and Overnight Blood BC were evaluated for their capacity to present α-GalCer or α-GalGalCer antigens to iNKT cells by the level of iNKT cell activation inferred by measuring cytokine release by the iNKT cells.

**Figure 2:**
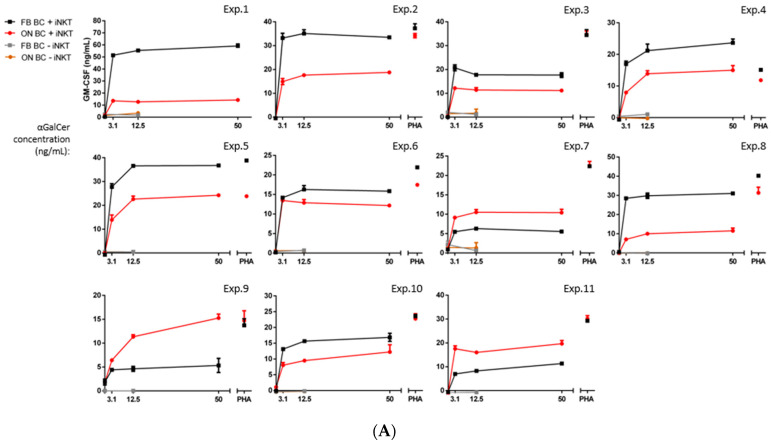

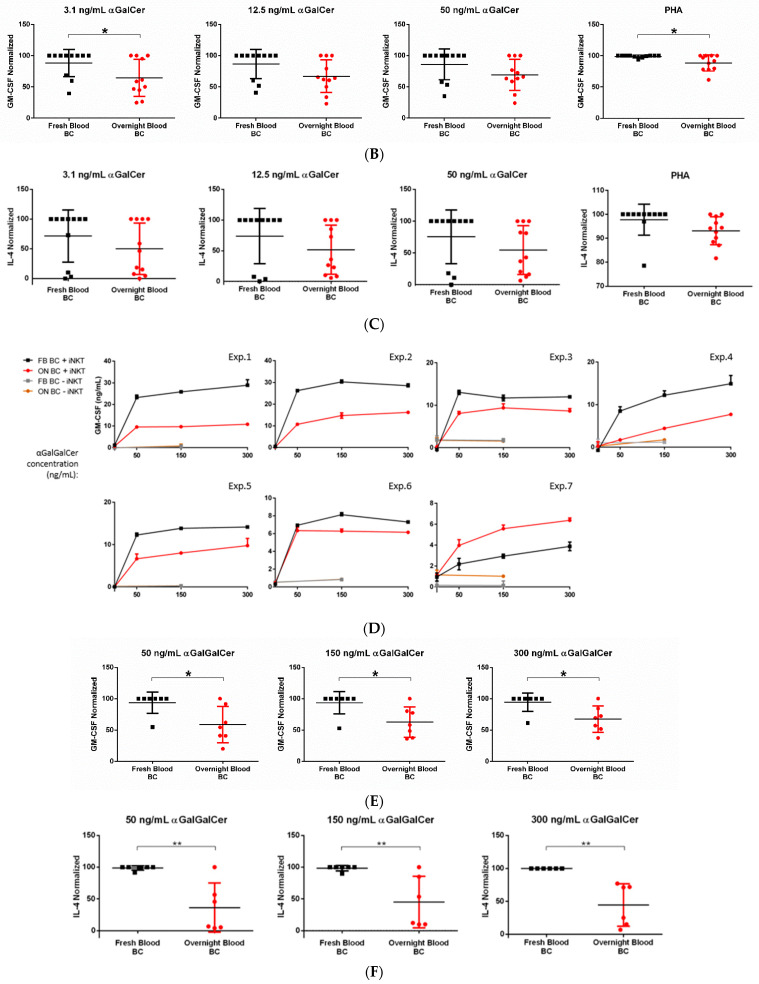
Monocytes isolated from Fresh Blood Buffy Coats have a higher capacity to present lipid antigens by CD1d than monocytes isolated from Overnight Blood Buffy Coats. Monocytes were incubated with 3.1, 12.5, and 50 ng/mL of α-GalCer, or 50, 150, and 300 ng/mL of α-GalGalCer, or 1 μg/mL PHA and cultured with and without iNKT cells. The iNKT cell response was analyzed by measuring GM-CSF and IL-4 release to the supernatant by ELISA. Black lines and square-shaped dots:Fresh Blood Buffy Cots (FB BC); red lines and circle dots:Overnight Blood BC (ON BC). (**A**) Eleven independent lipid antigen presentation assays with monocytes from Fresh Blood BC and Overnight Blood BC and α-GalCer as lipid antigen. Grey line with square dots corresponds to conditions of monocytes from Fresh Blood BC without iNKT cells, and brown circle dots represent conditions with monocytes from Overnight Blood BC without iNKT cells. Values represent the Mean ± SD of duplicates; (Exp.: experiment). (**B**) Normalization of the absolute values of GM-CSF from (**A**), individual values (points), and mean values (bar). Normalization was conducted considering 100 the highest concentration of GM-CSF in each independent experiment. (**C**) Normalization of the absolute values of IL-4 produced when using α-GalCer for each concentration and PHA. Normalization as in (**B**). (**D**) Seven independent lipid antigen assays with lipid antigen α-GalGalCer. Grey line with square dots, and brown circle dots corresponds to conditions with monocytes from Fresh Blood BC and Overnight Blood BC, respectively, without iNKT cells. Values represent the Mean ± SD of duplicates; (Exp.: experiment). (**E**) Normalization of the absolute values of GM-CSF produced when using α-GalGalCer for each concentration. Normalization as in (**B**). (**F**) Normalization of the absolute values of IL-4 produced when using α-GalGalCer for each concentration. Normalization as in (**B**). Statistical analysis was performed by *t*-test. * *p* < 0.05; ** *p* < 0.01.

In eight of the eleven experiments performed with α-GalCer, monocytes from the Fresh Blood BC had a higher capacity to activate iNKT cells than the monocytes from Overnight Blood BC, as assessed by the production of GM-CSF by iNKT cells (Figure 2A) and IL-4. Normalized values of produced GM-CSF induced by 3.1 ng/mL α-GalCer indicate that Fresh Blood BC monocytes have a statistically significant higher capacity to activate iNKT cells when compared to the values of Overnight Blood BC (*p*-value = 0.045) (Figure 2B). Normalized values of produced IL-4, however, do not show significant differences between BCs in the various concentrations of the lipid used; data seem to follow the tendency of the deduction stated previously (Figure 2C). Cytokine production was also measured in parallel cultures of monocytes only and monocytes plus α-GalCer without iNKT cells. As expected, monocyte culture without iNKT cells did not produce significant values of GM-CSF (Figure 2A), supporting the measurement of GM-CSF in the co-cultures as an indication of iNKT cell activation. Moreover, in parallel with the specific iNKT cell activation with α-GalCer, activation with Phytohemagglutinin (PHA) was performed. As shown in Figure 2A,B, iNKT cells in the presence of PHA and monocytes isolated from Fresh Blood BC are highly activated in the presence of PHA and monocytes from Overnight Blood BC.

Experiments with lipid antigen α-GalGalCer, which requires internalization and lysosomal processing before becoming antigenic, endorse the fact that monocytes from Fresh Blood BC have a higher capacity to activate iNKT cells (Figure 2D). Normalized values of GM-CSF reveal significant differences within all concentrations of α-GalGalCer tested (*p*-value 50 ng/mL = 0.017; *p*-value 150 ng/mL = 0.018; *p*-value 300 ng/mL = 0.016), confirming that monocytes derived from Fresh Blood BC show a higher capacity to activate iNKT cells (Figure 2E). Similarly, normalized values of IL-4 revealed significant differences in all concentrations of the lipid antigen tested (*p*-value 50 ng/mL = 0.0028; *p*-value 150 ng/mL = 0.0095; *p*-value 300 ng/mL = 0.0017) (Figure 2F).

### 3.2. Similar Viability, CD1d Expression, and Basal Activation of Monocytes Isolated from Fresh Blood and Overnight Blood Buffy Coats

**Figure 3:**
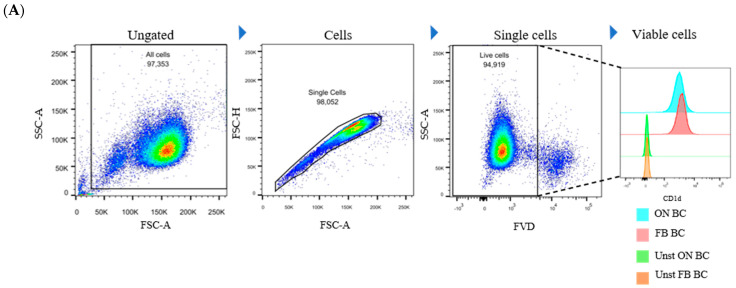

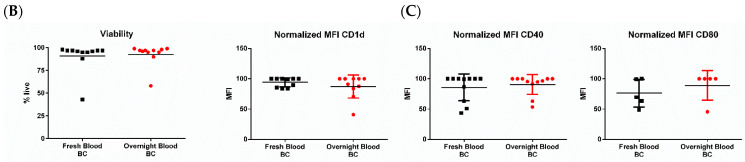
Monocyte viability; CD1d, CD40, and CD80 expression. (**A**) Representative example of gating strategy applied and of the histogram for CD1d expression with unstained samples in the last two peaks (orange and green) and stained samples in the upper peaks (red and blue). FB BC: Fresh Blood Buffy Coat; ON BC:Overnight Blood Buffy Coat; Unst ON BC:Unstained Samples from Overnight Blood BC; Unst FB BC:Unstained Samples from Fresh Blood BC. (**B**) Percentage of live cells in the experiments from the two types of Buffy Coat (BC). Monocyte viability was measured by flow cytometry using Fixable Viability Dye (FVD). (**C**) Normalization of the values of CD80, CD40, and CD1d MFI for each independent experiment. The values of MFI were relativized considering 100 as the highest value within each experiment for the chosen superficial marker. Statistical analysis was performed by *t*-test. Horizontal bars represent mean and vertical bars ± SD.

Monocyte viability, CD1d expression, and basal state of activation were assessed through flow cytometry using the gating strategy shown in Figure 3A. In all but one experiment, monocyte viability values were higher than 88% (Figure 3B). Viability was similar between monocytes isolated from Fresh Blood BC and Overnight Blood BC. Therefore, viability does not seem to justify the differences between the monocytes from the two types of BC in their capacities to present lipid antigens to iNKT cells.

In addition, it was investigated whether differences in CD1d expression (the antigen presenting molecule responsible for antigen presentation to iNKT cells) or basal activation state could account for the different capacities shown by monocytes from Fresh Blood BC versus Overnight Blood BC to present lipid antigens. Normalized values of MFI of CD1d, CD80, and CD40 were calculated after gating single cells and live cells and revealed no significant differences between the two BC generating protocols (Figure 3C).

The differences in lipid antigen presentation of monocytes isolated from Fresh Blood BC versus Overnight Blood BC are not associated with differences in viability or basal level of monocyte activation as measured by CD40 and CD80 expression. Similarly, the expression of CD1d is not affected by the different BC processing protocols.

The differences in monocytes’ functionality may be related to the fact that Fresh Blood BC cells stay less time in contact with the whole blood and its various cells and components, because the blood donation is processed on the same day of collection. Therefore, when compared to Overnight Blood BC, monocytes spend more time concentrated in the BC bag with some residual red blood cells before being analyzed. Thus, different residual blood components that proceed to the BC bag may have a role in monocyte functional differences observed. One of the candidates to influence monocyte function are red blood cells, which were shown to have a determinant role in potentiating T cell survival and proliferation [11]. More specifically, it was stated that red blood cells could function as modulators of T-cell apoptosis in vitro and, concurrently, in the reduction in intracellular oxidative stress [11]. Indeed, we can speculate that the contact of monocytes with residual red blood cells in a concentrated environment such as the BC bag could also have a potentiating effect in monocytes, namely in their function.

## 4. Conclusions

In summary, we show that in lipid antigen presentation assays monocytes from Fresh Blood BC demonstrated a higher capacity to activate iNKT cells than Overnight Blood BC, leading to the conclusion that functional differences exist in monocytes from different time-processed BCs. The BC continues to prove to be a reliable and regular/conventional source of blood cells, therefore this sample is of great importance in research dealing with blood components.

In conclusion, BC-processing protocols influence monocytes’ function in their capacity to present antigens. As such, the protocol for BC processing should be carefully considered in daily immunological activation assay research.

## Data Availability

The data presented in this study are available on request from the corresponding author. The data are not publicly available due to confidentiality of human subject data.
